# Growth Differentiation Factor-15 as an Emerging Biomarker in Cardiology: Diagnostic and Prognostic Implications

**DOI:** 10.3390/jpm16010016

**Published:** 2026-01-02

**Authors:** Carla Lombardi, Martina Marandola, Valentina Loria, Andrea Urbani, Silvia Baroni

**Affiliations:** 1 Unit of Chemistry, Biochemistry and Molecular Biology, “A. Gemelli” Hospital Foundation IRCCS, Largo A. Gemelli, 8, 00168 Rome, Italy; carla.lombardi@guest.policlinicogemelli.it (C.L.); andrea.urbani@policlinicogemelli.it (A.U.); 2 Department of Basic Biotechnological Sciences, Intensive Care and Perioperative Clinics, Catholic University of Sacred Heart, Largo F. Vito, 1, 00168 Rome, Italy; martina.marandola01@icatt.it; 3 Department of Emergency, Anesthesiological and Reanimation Sciences, “A. Gemelli” Hospital Foundation IRCCS, Largo A. Gemelli, 8, 00168 Rome, Italy; valentina.loria@policlinicogemelli.it

**Keywords:** GDF-15, cardiovascular disease, biomarker, heart failure, risk stratification

## Abstract

Growth Differentiation Factor-15 (GDF-15) is a stress-responsive cytokine belonging to the Transforming Growth Factor-beta (TGF-β) superfamily. Initially identified as macrophage inhibitory cytokine-1 (MIC-1), GDF-15 is expressed in various tissues and markedly upregulated under pathological conditions involving inflammation, oxidative stress, and tissue injury. Notably, GDF-15 upregulation has been associated with several cardiovascular events, such as heart failure, atrial fibrillation, atherosclerosis, coronary artery disease, and stroke. Furthermore, it has been observed that GDF-15, either alone or in combination with other cardiac biomarkers, can provide valuable complementary information enhancing risk assessment, early detection of cardiovascular events, and prediction of adverse outcomes. GDF-15 can be measured in various body fluids, using different methods. Immunoassays are widely employed and offer good sensitivity and reproducibility; however, variability between methods and potential interference from genetic variants highlight the need for standardization. This review summarizes current insights into GDF-15, with emphasis on its quantification methods, biological functions in cardiovascular diseases, and its emerging role as a diagnostic and prognostic biomarker.

## 1. Introduction

Cardiovascular diseases (CVDs) comprise a heterogeneous group of disorders affecting the heart and blood vessels, including coronary artery disease, heart failure, peripheral artery disease, and hypertension. According to the World Health Organization (WHO) [1], CVDs remain the leading cause of morbidity and mortality worldwide, accounting for approximately 17.9 million deaths each year. The complex pathophysiology of cardiovascular events frequently complicates early detection and precise risk stratification, thereby highlighting the need for biomarkers with significant diagnostic and prognostic value. In recent years, novel biomarkers have been identified, which allow for differentiation among diverse cardiac conditions and optimization of therapeutic strategies. Furthermore, the development of high-sensitivity assays has significantly improved the detection of established biomarkers, enabling earlier identification of cardiovascular events [2].

Among CVDs emerging biomarkers, several research focused on Growth Differentiation Factor–15 (GDF-15), a stress-responsive cytokine belonging to the Transforming Growth Factor-beta (TGF-β) superfamily. It was first identified by Bootcov et al. [3] as an autocrine cytokine secreted by activated macrophages and was therefore initially named macrophage inhibitory cytokine-1 (MIC-1). As its pleiotropic nature and tissue-specific functions became evident, GDF-15 has been referred to by several other names over time, including nonsteroidal anti-inflammatory drug-activated gene-1 (NAG-1), placental bone morphogenetic protein (PLAB), placental transforming growth factor-β (PTGFB), and prostate-derived factor (PDF) [4,5].

GDF-15 is expressed in a broad range of physiological and pathological contexts, highlighting its multifaced role as both a cellular stress biomarker and a modulator of systemic, metabolic and inflammatory responses.

In recent years, multiple studies demonstrated that GDF-15 levels are associated with the adverse cardiovascular events across a spectrum of CVD conditions including heart failure, chest pain, acute coronary syndrome, stable ischemic heart disease, stroke and atrial fibrillation [6].

However, despite this expanding literature, the clinical interpretation and translational applicability of GDF-15 in cardiovascular medicine remain incompletely defined.

In light of these considerations, the aim of the present review is to offer a focused and critical description of the current evidence supporting the diagnostic and prognostic value of GDF-15 in cardiovascular diseases.

## 2. Methods

This review includes Pubmed-indexed articles, written in English and published between 2000 and 2025. Studies published before 2000 were considered only if they were of particular historical or methodological relevance, such as early pioneering work on GDF-15. The database was queried using the following keywords, either individually or in combination: GDF-15, cardiovascular disease, biomarker, heart failure, risk stratification, acute coronary syndrome, myocardial infarction, atherosclerosis, stroke, atrial fibrillation, prognosis, diagnosis, mortality. The keywords were combined using Boolean operators (AND, OR) to refine the search strategy. Data extracted from the selected studies focused on GDF-15 structure and functions, measurement methods, and its applications in cardiology settings.

## 3. Synthesis and Expression

Located on chromosome 19p12–13.1, the GDF-15 gene (ID9518) exhibits a two-exon structure (309 bp and 891 bp) separated by a 2.9 kb intron [7,8].

**Figure 1:**
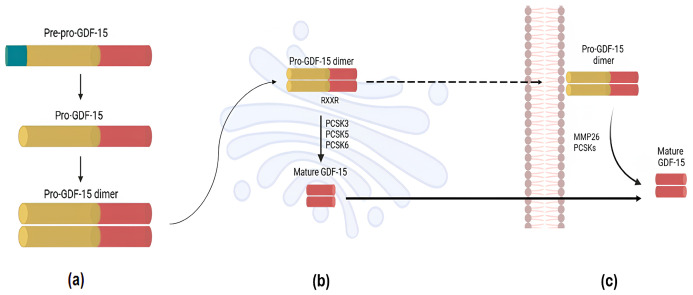
Schematic representation of GDF-15 biosynthesis, maturation, and secretion. (**a**) GDF-15 is synthesized as pre-pro-GDF-15, consisting of a signal peptide (blue), a pro-domain (yellow), and a mature C-terminal domain (red). Removal of the signal peptide generates pro-GDF-15, which rapidly dimerizes; (**b**) In the Golgi apparatus, pro-GDF-15 dimer is cleaved by PCSK3, PCSK5, and PCSK6 at the RXXR site, producing mature GDF-15; (**c**) Mature GDF-15 homodimers are secreted through the classical secretory pathway and released into circulation, while in specific cells pro-GDF-15 dimers may remain bound to the extracellular matrix until further cleavage by MMP26 or PCSKs. GDF-15: Growth Differentiation Factor-15; PCSK: proprotein convertase subtilisin–kexin; MMP26: matrix metalloproteinase 26.

The protein is initially synthesized as an inactive propeptide, known as pre-pro-GDF-15, composed of 308 amino acids (aa) and with a molecular weight of approximately 35 kDa [9]. This precursor comprises an N-terminal signal peptide of 29 aa, with storage and trafficking functions [10]; a 167 aa pro-domain; and a C-terminal portion of 112 amino acids, which represents the mature form of the protein [11]. The signal peptide is then removed with a proteolytic cleavage, generating pro-GDF-15 (~30 kDa) which rapidly dimerizes [12] (Figure 1a). β-arrestin 1 mediates the translocation of pro-GDF-15 dimers to the Golgi apparatus, where they undergo sequential proteolytic processing by proprotein convertase subtilisin–kexin type 3 (PCSK3), PCSK 5 and PCSK 6 [9,13], which recognize the RXXR cleavage site located between the propetide sequence and the mature domain [14] (Figure 1b). After complete processing, GDF-15 is secreted as a biologically active homodimer, in which the two molecules are linked by a disulfide bond [15,16].

This bond contributes to the conformational stability of the protein and occurs within a sequence of seven cysteine residues, which is conserved among members of the TGF-β family [10,17].

The secretion of GDF-15 occurs through the classical secretory pathway, in which the mature protein is packaged into vesicles within the Golgi apparatus and subsequently released into the extracellular space via exocytosis [18,19] (Figure 1c).

Given its lower affinity to the extracellular matrix (ECM), the homodimeric mature form is released into the systemic circulation [19,20]. In specific types of cells or under pathological conditions, the unprocessed form of the protein may also be secreted, although it does not enter systemic circulation [21]. For example, cancer cells and placental cytotrophoblasts secrete pro-GDF-15 dimers which remain anchored to the ECM until the pro-domain is cleaved by the matrix metalloproteinase 26 (MMP26) and PCSKs, respectively [12,22] (Figure 1c).

In healthy individuals, circulating GDF-15 levels are relatively low, with values ranging between 200 and 1200 pg/mL in adults [23,24], with expression primarily observed in kidney [25], intestine [26], liver [27], and placenta [28]. A minimal GDF-15 expression is observed in other organs as well including gallbladder, pancreas, and lung [29,30]. In physiological conditions, GDF-15 levels follow a circadian rhythm, slowly increase with aging and seem to be less influenced by sex or ethnicity [31,32]. However, in cellular stress conditions or pathological states GDF-15 expression is markedly upregulated.

Increased GDF-15 levels have been observed in association with diabetes [12], smoking [33], surgical stress [34], intense physical activity [35], cancer [36], non-alcoholic fatty liver disease (NAFLD) [37], as well as renal diseases [38].

Of note, GDF-15 upregulation has been demonstrated after various cardiovascular events triggering inflammation, endothelial activation and oxidative stress, including pressure overload, heart failure (HF), atrial fibrillation and atherosclerosis [39,40,41]. In these contexts, GDF-15 is abundantly expressed and secreted by a variety of cell types: macrophages, vascular smooth muscle cells (VSMCs), endothelial cells (ECs), adipocytes, and cardiomyocytes [42]. Interestingly, GDF15-specific genetic variants have been shown to cause phenotypic variation in circulating GDF-15 concentrations [8], suggesting that such polymorphisms may not only influence baseline GDF-15 expression but also modulate the individual susceptibility and response to cardiovascular stress.

## 4. Regulation and Signaling

**Figure 2:**
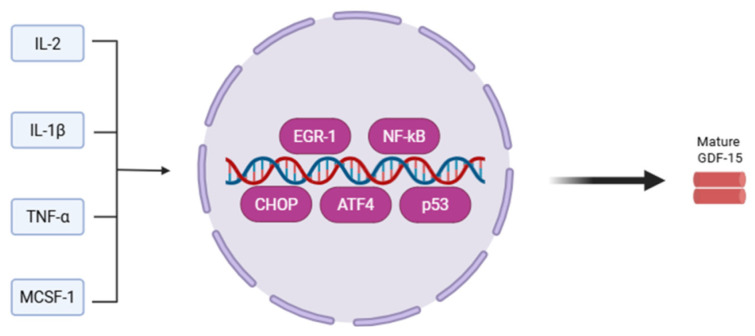
Molecular regulation of GDF-15. Inflammatory and stress-related mediators (IL-1β, TNF-α, IL-2, M-CSF) regulate GDF-15 expression by activating key transcription factors such as ATF4, CHOP, p53, EGR-1, and NF-κB, emphasizing the complex and tissue-dependent nature of this regulatory network. IL-1ß: interleukin-1ß; TNF-α: tumor necrosis factor-α, IL-2: interleukin-2; MCSF-1: macrophage colony-stimulating factor-1; EGR-1: early growth response transcription factor 1; NF-Kb: Nuclear Factor kappa B; CHOP: C/EBP homologous protein; ATF4: activating transcription factor 4; GDF-15: Growth Differentiation Factor-15.

As a stress-inducible cytokine, GDF-15 is regulated by several inflammatory or stress-related proteins such as interleukin (IL)-1ß, tumor necrosis factor (TNF)-α, IL-2, and macrophage colony-stimulating factor (MCSF)-1, suggesting a complex and tissue-specific regulation [43]. These molecules have been shown to promote GDF-15 expression through an intricate network of signaling pathways resulting in the activation of: activating transcription factor 4 (ATF4), C/EBP homologous protein (CHOP), p53, early growth response transcription factor 1 (EGR-1), and Nuclear Factor kappa B (NF-kB) [14,44] (Figure 2).

Mature GDF-15 exerts its effects through the glial cell-derived neurotrophic factor (GDNF) family receptor α-like (GFRAL), which is specifically expressed in the hindbrain neurons [45]. Upon dimerization, GFRAL promotes the recruitment of rearranged during transfection (RET) tyrosine kinase, which acts as coreceptor [13]. This interaction subsequently triggers downstream signaling cascades, including the phosphorylation of extracellular signal-regulated kinase (ERK), the proto-oncogene c-Fos, phosphoinositide phospholipase C1 (PLC1), and protein kinase B (PKB) [29].

**Figure 3:**
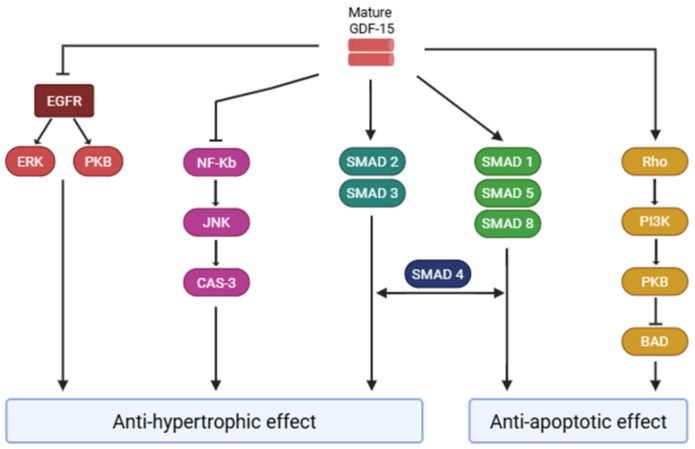
GDF-15–mediated mechanisms of cardiac protection. In cardiomyocytes, GDF-15 activates the Smad2/3 and Smad1/5/8 pathways to limit hypertrophy and apoptosis, enhances myocardial survival through the PI3K/PKB signaling cascade, and inhibits pro-apoptotic pathways involving JNK, Bad, and EGFR, thereby contributing to cardiac protection. GDF-15: Growth Differentiation Factor-15; EGFR: epidermal growth factor receptor; ERK: extracellular signal-regulated kinase; PKB: protein kinase B; NF-Kb: Nuclear Factor kappa B; JNK: c-Jun N-terminal kinase; CAS-3: Caspase-3; PI3K: phosphatidylinositol 3-kinase; BAD: Bcl-2-associated death promoter.

Importantly, multiple lines of evidence suggest that GDF-15 may also exert effects in peripheral tissues, including the heart [46] (Figure 3). In fact, experimental data demonstrated how GDF-15 is able to activate the Smad 2/3 and the Smad 1/5/8 signaling pathways in cultured cardiomyocytes, reducing hypertrophy and apoptosis during heart failure [47].

Moreover, there is evidence that GDF-15 may contribute to myocardial preservation by activating the signaling pathways mediated by phosphatidylinositol 3-kinase (PI3K) and PKB in rat cardiomyocytes [48].

Finally, GDF-15 is thought to act as a cardioprotective factor by inhibiting c-Jun N-terminal kinase (JNK), Bcl-2-associated death promoter (Bad), and epidermal growth factor receptor (EGFR) [49].

Of note, it seems that the increase in GDF-15 levels in ischemia–reperfusion cellular models is associated with cardioprotective effects [50].

Similarly, recombinant GDF-15 administration reduces myocardial damage and inflammation [51].

## 5. Measurements and Methods

GDF-15 is detectable in multiple human biological fluids, including blood, urine and seminal plasma, with varying concentration depending on the specific biological matrix and the underlying physiological or pathological conditions. Although seminal plasma and urine can provide valuable insights in specific settings [52,53,54], serum and plasma are the most widely used matrices for quantitative GDF-15 assessment.

All currently commercially available assays for GDF-15 quantification are based on immunometric principles employing different detection methods: enzyme-linked immunosorbent assay (ELISA), cytometric bead array, electrochemiluminescence immunoassay (ECLIA), and chemiluminescence immunoassay (CLIA) (Table 1).

Most GDF-15 assays are intended exclusively for research use, with only the Roche Elecsys ECLIA having received approval for in vitro diagnostic (IVD) applications, highlighting the limited availability of clinically validated assays for this biomarker.

In 2007, Kempf et al. developed a new immunoradiometric sandwich assay (IRMA) for the detection of circulating GDF-15, showing a sensitivity of 20 pg/mL [67]. This method has been used in many studies exploring GDF-15 levels in different cardiovascular settings, showing good sensitivity and specificity [68,69,70].

In 2017, a multicenter study by Wollert et al. evaluated the ECLIA Elecsys GDF-15 assay (Roche Diagnostics), comparing it with the IRMA method developed by Kempf et al. and the ELISA Quantikine assay (R&D Systems). The results showed that while the Elecsys assay offers excellent precision and reproducibility, the absolute GDF-15 concentrations obtained may differ substantially from those measured using other analytical techniques [71].

These discrepancies highlight the critical need for analytical standardization to ensure consistency, accuracy, and comparability of GDF-15 measurements across populations and clinical settings. Moreover, recent evidence suggests that part of the variability in measured GDF-15 levels may not be purely methodological. Interestingly, it has been demonstrated that the genetic variant H202D alters GDF-15 antigenicity, potentially inducing antibody cross-reactivity in some ELISA measurements and thereby interfering with its quantification [72,73,74]. This finding implies that individual genetic differences may influence the accuracy and comparability of GDF-15 quantification.

In this regard, mass spectrometry could represent a strategy to overcome antibody cross-reactivity, improving the accuracy of circulating GDF-15 quantification. For example, Lancrajan et al. [75] developed a protocol based on partial denaturation of serum proteins by organic solvents, combined with size exclusion chromatography. The absolute quantification of GDF-15 was performed by liquid chromatography electrospray ionization mass spectrometry (LC-ESI MS), using isotope labeled and concatamerized fingerprint peptides. GDF-15 quantification by this protocol has been shown to correlate well with ELISA measurements, providing higher resolution at low biomarker concentrations compared to antibody-based assays, while also enabling multiplexed analysis.

However, mass spectrometry-based approaches are generally more technically complex, time-consuming, and costly than conventional immunoassay. They also require specialized instrumentation and expertise, and pre-analytical steps such as sample enrichment and digestion can introduce variability, limiting their applicability in large-scale clinical studies. As a result, the potential of mass spectrometry for GDF-15 quantification remains poorly explored, highlighting the need for further research.

## 6. GDF-15 and Cardiovascular Diseases

Although its molecular mechanisms are not yet fully elucidated, preclinical studies using cellular systems and animal models suggest that increased GDF-15 expression may exert cardioprotective effects, particularly in response to cardiac stress and injury [50,51]. These experimental findings indicate a potential adaptive role for GDF-15 in limiting inflammation, cellular damage, and adverse cardiac remodeling. However, such protective effects have not been consistently confirmed in clinical studies. Elevated circulating levels of GDF-15 in CVDs patients are commonly associated with an increased risk of cardiovascular events, disease severity, and poor clinical outcomes, thus conferring a negative prognostic value. In the following sections, the role of GDF-15 will be described in detail across different cardiovascular conditions.

### 6.1. Heart Failure (HF)

Heart failure (HF) is a clinical syndrome in which the heart is unable to pump sufficient blood to satisfy the body’s circulatory demands, resulting in symptoms such as shortness of breath and fatigue. It can develop because of various disorders affecting the myocardium or the heart valves. Heart failure occurs with roughly equal frequency in patients with reduced and preserved ventricular ejection fraction, each accounting for about 50% of all cases [76].

Elevated circulating levels of GDF-15 have been consistently linked to adverse cardiovascular outcomes, including an increased risk of heart disease, heart failure, and mortality following both ST-segment elevation and non-ST-elevation myocardial infarction [6,77]. Unlike conventional biomarkers such as B-type natriuretic peptides, which primarily reflect hemodynamic stress, GDF-15 provides complementary information about systemic and cellular stress responses. Studies directly comparing the predictive significance of GDF-15 with traditional biomarkers such as N-terminal pro-B-type natriuretic peptide (NT-proBNP) and high-sensitivity troponin T (hs-TnT), or incorporating GDF-15 into risk scores [78,79,80], demonstrated that it improved the prediction of adverse outcomes in patients with HF. Interestingly, Kosum et al. observed that GDF-15 levels significantly declined throughout hospitalization in patients with acute heart failure (AHF), and lower discharge GDF-15 levels were associated with a lower risk of 30-day rehospitalization, supporting its value as a dynamic marker reflecting the course of AHF with a great prognostic power [81].

Furthemore, GDF-15 emerged as the strongest predictor of HF before the first hospitalization [82]. Studies in the pre-hospitalization phase are particularly valuable, as identifying patients at risk of adverse HF outcomes at an early stage may yield substantial clinical, societal, and economic benefits. Early detection allows intervention when underlying disease mechanisms are still modifiable, preserving the overall health status, and the high financial burden associated with HF hospitalization has not yet occurred. As the population ages and cardiometabolic comorbidities predisposing to HF become increasingly common, early risk stratification will become even more important to enable preventive strategies [83].

In this context, Bradley et al. observed that in patients at risk of HF or with HF prior to their first hospitalization, GDF-15 offers unique prognostic information and serves as a powerful predictor of HF hospitalization (HFH) and all-cause mortality [83].

### 6.2. Atherosclerosis

During the progression of atherosclerosis, GDF-15 expression is markedly increased, particularly within macrophages of atherosclerotic plaques in both humans and mice [12].

One of the first studies investigating GDF-15 in atherosclerosis was conducted in 2011. De Jager et al. identified that GDF 15 deficiency exerts protective effects in atherosclerosis by reducing C-C chemokine receptor 2 (CCR2)-mediated macrophage chemotaxis and modulating cell death. Hematopoietic GDF 15 deficiency limits early lesion formation and enhances plaque stability through increased collagen deposition and reduced necrotic core expansion [84].

Another study investigated the advanced atherogenic lipid profile using the Liposcale^®^ test (Biosfer Teslab, Reus, Spain), highlighting that increased GDF-15 plasma concentrations are associated with a more atherogenic lipid profile. Specifically, GDF-15 increased levels were correlated to elevated Very Low-Density Lipoproteins (VLDL), Low-Density Lipoprotein (LDL) cholesterol and triglyceride content, a higher number of small and dense lipoprotein particles, and reduced High-Density Lipoproteins (HDL) cholesterol. These results indicate that elevated GDF-15 levels may reflect or contribute to lipid abnormalities that promote atherosclerosis and cardiovascular risk [85].

Park et al. reported significantly elevated circulating levels of GDF-15 in patients with atherosclerotic disease undergoing surgical revascularization, suggesting that it could facilitate earlier risk factor management to prevent disease onset and progression [86].

Furthermore, Guardiola et al. observed that GDF-15-specific genetic variants may contribute as well to the development of atherosclerosis. Specifically, carriers of the rs1054564 variant exhibited a higher prevalence of carotid atherosclerotic plaques compared with wild-type individuals [87]. In this specific case, GDF-15 serum levels were associated with diabetes and subclinical atherosclerosis in carriers of the rs1054564 variant, but no significant associations were observed with other metabolic parameters, including hypertension, obesity, metabolic syndrome, or liver steatosis [87].

### 6.3. Coronary Artery Disease (CAD)

Coronary artery disease (CAD) is one of the most prevalent cardiovascular disorders globally and continues to represent a leading cause of morbidity and mortality in both industrialized and developing countries. Its pathogenesis involves a multifactorial interplay among lifestyle, environmental, and genetic components. Among the principal determinants associated with CAD are diabetes mellitus, arterial hypertension, smoking, dyslipidemia, obesity, elevated homocysteine levels, and psychosocial stress [88].

In the context of CAD, elevated GDF-15 levels have been linked to worse clinical outcomes, including increased risks of myocardial infarction (MI), cardiovascular death, and all-cause mortality. However, high GDF-15 concentrations showed limited predictive value for myocardial infarction (MI) in patients with CAD, particularly in those with acute coronary syndrome (ACS). Only at the highest concentrations did the difference in MI risk become more apparent compared with the lowest GDF-15 levels, highlighting a concentration-dependent prognostic utility [89].

Multiple studies have shown that increased GDF-15 levels are predictive of poor prognosis in CAD patients. For instance, a meta-analysis involving over 28,000 stable CAD patients found that the highest GDF-15 levels were linked to a 42% increased risk of major adverse cardiovascular events, a 64% higher risk of cardiovascular mortality, and a 101% increased risk of all-cause mortality compared to the lowest levels [90]. The prognostic significance of GDF-15 is not limited to acute events: in stable CAD patients, higher GDF-15 levels have been found to be predictive of future cardiovascular events and mortality, independently of other established biomarkers such as N-terminal pro B-type natriuretic peptide (NT-proBNP) and high-sensitivity C-reactive protein (hs-CRP) [91]. Additionally, GDF-15 concentrations correlate with the extent of CAD, as measured by the Gensini score, and have shown high sensitivity and specificity for disease detection [92].

Nevertheless, in the context of CAD the role of GDF-15 still needs to be further elucidated.

### 6.4. Atrial Fibrillation (AF)

Atrial fibrillation (AF) is the most prevalent cardiac arrhythmia and represents a substantial burden on healthcare systems. AF incidence rises sharply with age, making it particularly common in elderly populations. It is also recognized as a major risk factor for thromboembolic events, including ischemic stroke, as well as for heart failure and overall mortality [93].

GDF-15 has emerged as a promising biomarker in this context. Elevated GDF-15 levels have been associated with adverse outcomes in AF patients, including increased risks of mortality, major adverse cardiac events (MACE), and thromboembolic complications. In a prospective cohort study involving 362 patients with non-valvular AF, GDF-15 levels were independently associated with all-cause mortality and MACE. Patients with higher GDF-15 concentrations had significantly lower survival probabilities over six years compared to those with lower levels, indicating its potential utility as a prognostic indicator in AF management [94].

Moreover, in patients with AF and rheumatic heart disease, elevated GDF-15 levels correlated with increased atrial fibrosis, suggesting a potential role for GDF-15 in the structural remodeling of the atria that underlies AF development and persistence [95].

### 6.5. Stroke

Elevated circulating GDF-15 levels have been consistently associated with acute ischemic stroke (AIS), correlating with worse functional outcomes and higher mortality. Clinical studies show that higher baseline GDF-15 predicts adverse events at 3 months post-stroke, independent of traditional risk factors, age, and stroke severity [96].

Elevated circulating levels of GDF 15 in the acute phase of ischemic stroke have been independently associated with a higher incidence of post stroke depression (PSD) at three months, suggesting that GDF-15 could be an important prognostic marker for identifying patients at higher risk of neuropsychiatric sequelae [97].

Furthermore, according to Brenière et al., admission plasma GDF-15 levels independently predict three-month mortality in ischemic stroke patients treated with acute revascularization. Deceased patients had a median GDF-15 of 2777 pg/mL, significantly higher than the 1460 pg/mL observed in survivors [98].

In hypertensive patients without prior stroke, higher plasma GDF-15 concentrations are correlated to an increased risk of first-ever ischemic stroke over follow-up periods of several years [99].

According to Alshehri et al., metformin enhances GDF-15 signaling, which mitigates inflammatory and oxidative stress responses in ischemic stroke. Targeting this pathway with selective activators may represent a promising strategy for managing both acute and chronic stroke [100].

These findings suggest that GDF-15 not only reflects ongoing cerebral injury but may also serve as a prognostic biomarker to identify high-risk patients and guide clinical management.

However, the predictive value of GDF-15 for stroke remains uncertain, and current evidence is insufficient to establish a clear association [89]. In many contexts, limited numbers of recurrent events or hemorrhagic complications reduce the power to detect meaningful associations, including the potential link between high GDF-15 levels and bleeding risk. Furthermore, incomplete mortality data and the observational nature of most investigations make it challenging to draw firm causal conclusions [98].

### 6.6. Cardiovascular Surgery

Cardiovascular surgery remains a pivotal element in the management of advanced cardiac and great vessel diseases, including procedures such as coronary artery bypass grafting (CABG), surgical and transcatheter valve replacement, and corrective operations for aortic pathology [101].

The field has considerably evolved, driven by an aging population, rising prevalence of cardiovascular risk factors, and rapid technological advances that allow for less invasive approaches and more complex reconstructions [102].

Despite improvements in perioperative care and risk stratification, surgical interventions still carry significant morbidity and mortality risks, making the identification of novel biomarkers and personalized strategies crucial for optimizing outcomes [34].

Ferreira et al. showed that in patients undergoing isolated surgical aortic valve replacement, GDF-15 levels peaked at 6 h post-surgery and were positively correlated with cardiopulmonary bypass and aortic cross-clamp times. Additionally, GDF-15 levels correlated with postoperative Sequential Organ Failure Assessment (SOFA) scores at 24 h after surgery, suggesting its potential utility in early risk assessment and in helping guide treatment choices for patients undergoing cardiac surgery [103].

Moreover, GDF-15 has been identified as a valuable marker for predicting long-term outcomes in cardiovascular interventions. In patients undergoing transcatheter aortic valve replacement (TAVR), increased baseline GDF-15 concentrations were linked to higher 1-year mortality rates and lack of reverse remodeling. Incorporating GDF-15 measurements into the Society of Thoracic Surgeons risk score improved the predictive accuracy for mortality, highlighting its potential role in enhancing risk assessment models for cardiovascular surgical procedures [104].

## 7. GDF-15 and Other Cardiovascular Biomarkers

Even though GDF-15 could represent a versatile biomarker, its clinical utility requires careful evaluation of both its strengths and limitations. This becomes particularly important when compared with biomarkers like Soluble Suppression of Tumorigenicity 2 (sST2), which reflects myocardial adverse remodeling [105], and NT-proBNP, the standard indicator of ventricular wall stretch and hemodynamic overload [106].

NT-proBNP remains the reference biomarker in heart failure, displaying a central role in screening, diagnosis, prognostic stratification, and clinical monitoring [107]. Moreover, its role in ruling out heart failure in the emergency department is well established, supported by the definition of specific age-adjusted cut-off values [108]. Furthermore, large cohort analyses show that elevated NT-proBNP levels are independently associated with higher risk of heart failure hospitalization and cardiovascular death, and that it contributes more strongly to risk prediction models than GDF-15 in diverse patient populations, including those with atrial fibrillation and heart failure risk [107].

Serial NT-proBNP measurements further improve risk assessment, reflecting changes in cardiac stress over time [109]. Despite being a highly specific marker of cardiac mechanical stress, NT-proBNP is characterized by high intra-individual biological variability [110]. In this context, other biomarkers such as sST2 and GDF-15, although less specific for cardiac stress, may help to better characterize HF patients with a more complex pathophysiological profile. Both sST2 and GDF-15 exhibit lower intra-individual biological variability compared with natriuretic peptides, but they have reduced specificity because their circulating levels are strongly influenced by systemic and local inflammatory processes [111,112]. However, this apparent limitation may represent an advantage, as these biomarkers could assist clinicians in capturing the multifactorial complexity that characterizes heart failure.

Importantly, both sST2 and GDF-15 have been shown to significantly improve prognostic assessment in heart failure patients, but they provide complementary information over different time frames. Kuster et al. observed that sST2 is primarily associated with short-term outcomes in HF, with prognostic relevance generally limited to approximately one year. In contrast, GDF-15 appears to reflect mid- to long-term risk, offering sustained prognostic information for up to five years and showing lower sensitivity to heart failure therapies. Accordingly, sST2 may be particularly useful for guiding short-term treatment decisions, whereas GDF-15 could help identify patients at risk for long-term adverse events, thereby supporting personalized long-term management strategies, including tailored rehabilitation programs [112]. Overall, a multimarker approach may provide a comprehensive assessment of HF patients, integrating hemodynamic information derived from NT-proBNP with pathophysiological insights obtained from sST2 and GDF-15. Therefore, despite its promising role, further studies are needed to better define the clinical utility of GDF-15 in cardiovascular settings. In this regard, the use of automated ECLIA platforms for GDF-15 measurement could yield more robust and reproducible results, facilitating real-time comparison with traditional biomarkers in routine clinical practice.

## 8. Conclusions

GDF-15 is a versatile biomarker that reflects a variety of pathophysiological processes, such as inflammation, oxidative stress, tissue damage, and metabolic imbalances. Initially recognized as a stress-responsive cytokine, GDF-15 has emerged as a comprehensive marker of cardiovascular risk and disease progression across a wide range of conditions, including heart failure, atherosclerosis, coronary artery disease, atrial fibrillation, and stroke. The main CVDs-related findings are summarized in Table 2.

The strong association between elevated levels of circulating GDF-15 and adverse cardiovascular outcomes highlights its potential as a valuable prognostic biomarker across various stages of disease (from early risk assessment to outcomes after hospitalization).

Moreover, GDF-15 has shown potential in enhancing existing risk models. Studies have demonstrated that incorporating GDF-15 improves the predictive accuracy of heart failure risk scores, provides better assessments of bleeding risk in atrial fibrillation, and offers additional prognostic insights in coronary and valvular diseases. Its dynamic variation in acute contexts, such as the observed decrease during hospitalization for acute heart failure, further underscores its value as a real-time marker of treatment response.

NT-proBNP remains the cornerstone biomarker in cardiovascular disease owing to its strong diagnostic and prognostic value, wide availability, and extensive clinical validation; however, its interpretation is limited by significant biological variability and confounding by age, renal function, and body mass index. sST2 provides complementary prognostic information, particularly in heart failure and myocardial stress, with lower intraindividual variability and reduced influence of renal function and its role in routine clinical decision-making is still evolving. In contrast, GDF-15 emerges as a robust marker of systemic cellular stress with low intraindividual biological variability and strong prognostic associations across cardiovascular conditions, although its limited disease specificity and fewer interventional data currently restrict its standalone clinical application.

Moreover, further studies are needed to clarify the apparent discrepancy between preclinical models, in which GDF-15 seems to exert cardioprotective effects, and clinical observations, where elevated circulating GDF-15 is consistently associated with adverse outcomes and to elucidate the apparently significant role of GDF-15 in the pathophysiology of cardiovascular diseases.

In conclusion, GDF-15 represents a valuable candidate for multimarker strategies aimed at improving cardiovascular risk prediction and personalized patient care. The increasing availability of fully automated ECLIA-based assays may facilitate real-time comparison with established cardiovascular biomarkers, improve analytical harmonization, and enable the investigation of GDF-15 across diverse and well-characterized populations.

## Figures and Tables

**Table 1:** Commercially available GDF-15 assays.

Assay	Manufacturer	Method	Sensitivity (pg/mL)	Measurement Range (pg/mL)
Elecsys GDF-15 [55]	Roche Diagnostics	ECLIA	400	400–20,000
Human GDF15 CLIA Kit [56]	Elabscience Biotechnology Inc.	CLIA	4.69	7.81–500
ProcartaPlex Human GDF-15 Simplex Kit [57]	Invitrogen	Bead-based	~9.65	12.21–50,000
Human GDF15/MIC-1/PTGFB [58]	AimPlex Biosciences Inc.	Bead-based	2	5–5000
Quantikine Human GDF-15 ELISA Kit [59]	R&D Systems	ELISA	4.39	23.4–1500
Human GDF-15 ELISA Kit [60]	Invitrogen	ELISA	2	1.1–800
Human GDF-15 ELISA Kit [61]	Elabscience Biotechnology Inc.	ELISA	14.06	23.44–1500
Human GDF-15 ELISA Kit [62]	FineTest	ELISA	14.063	23.44–1500
Human GDF-15 ELISA Kit [63]	Proteintech	ELISA	300	1250–40,000
Human GDF-15 ELISA Kit [64]	ACRO Biosystems	ELISA	7	11.72–1500
Human GDF-15 ELISA Kit [65]	Abcam	ELISA	2	1.1–800
Human GDF-15/MIC-1 ELISA Kit [66]	Sigma-Aldrich	ELISA	2	1.1–800

GDF-15: Growth differentiation factor-15; GDF15: Growth differentiation factor 15; ECLIA: Electrochemiluminescence Immunoassay; CLIA: Chemiluminescence Immunoassay; MIC-1: Macrophage inhibitory cytokine-1; PTGFB: Placental transforming growth factor-β; ELISA: enzyme-linked immunosorbent assay.

**Table 2:** Clinical findings related to GDF-15 and cardiovascular diseases.

Condition	GDF-15	References
Heart failure (HF)	High levels associated with adverse cardiovascular outcomes, increased mortality, and higher risk of hospitalization. Provides additional prognostic value compared to NT-proBNP and hs-TnT. Decreases during hospitalization in acute heart failure (AHF), improving prediction of 30-day rehospitalization. Strong predictor of heart failure before the first hospitalization.	[6,76,77,78,79,80,81,82,83]
Atherosclerosis	Increased expression in atherosclerotic plaques, especially in macrophages. Deficiency reduces early lesions, improves plaque stability, and limits necrotic core expansion. Elevated circulating levels associated with a more atherogenic lipid profile. Higher levels observed in patients with atherosclerotic disease compared to healthy individuals. Genetic variants (rs1054564) linked to higher prevalence of carotid plaques.	[12,84,85,86,87]
Coronary Artery Disease (CAD)	Elevated levels predict myocardial infarction (MI), cardiovascular death, and all-cause mortality. Elevated levels increase risk for major adverse cardiovascular events (MACE). Correlates with CAD severity (e.g., Gensini score). Prognostic value independent of NT-proBNP and hs-CRP.	[88,89,90,91,92]
Atrial Fibrillation (AF)	High levels predict mortality, MACE, and thromboembolic complications. Independent association with worse 6-year survival. Correlated with the degree of atrial fibrosis.	[93,94,95]
Stroke	High levels are associated with poor prognosis and higher 3-month mortality. Predicts post-stroke depression (PSD). Prognostic value in patients treated with acute revascularization. Elevated levels in hypertensive patients without prior stroke predict the first ischemic event.	[96,97,98,99,100]
Cardiovascular surgery	Peaks at 6 h after surgery and correlates with bypass time, cross-clamp duration, and SOFA score at 24 h. High baseline levels in TAVR patients are associated with higher 1-year mortality and lack of reverse remodeling. Improves the predictive accuracy for mortality if added to preoperative scores.	[34,101,102,103,104]

GDF-15: Growth Differentiation Factor-15; HF: Heart failure; NT-proBNP: N-terminal pro-B-type natriuretic peptide; hs-TnT: high-sensitivity troponin T; AHF: Acute heart failure; CAD: Coronary artery disease; MI: myocardial infarction; MACE: major adverse cardiovascular events; hs-CRP: high-sensitivity C-reactive protein; AF: atrial fibrillation; PSD: post-stroke depression; SOFA: Sequential Organ Failure Assessment; TAVR: transcatheter aortic valve replacement.

## Data Availability

No new data were created or analyzed in this study. Data sharing is not applicable to this article.

## Abbreviations

The following abbreviations are used in this manuscript:

GDF-15	Growth Differentiation Factor–15
TGF-β	Transforming Growth Factor-β
MIC-1	macrophage inhibitory cytokine-1
CVDs	cardiovascular diseases
WHO	World Health Organization
NAG-1	nonsteroidal anti-inflammatory drug-activated gene-1
PLAB	placental bone morphogenetic protein
PTGFB	placental transforming growth factor-β
PDF	prostate-derived factor
aa	amino acids
PCSK	proprotein convertase subtilisin–kexin
ECM	extracellular matrix
MMP26	matrix metalloproteinase 26
NAFLD	non-alcoholic fatty liver disease
HF	heart failure
VSMCs	vascular smooth muscle cells
ECs	endothelial cells
IL-1β	interleukin-1ß
TNF-α	tumor necrosis factor-α
IL-2	interleukin-2
MCSF-1	macrophage colony-stimulating factor-1
ATF4	activating transcription factor 4
CHOP	C/EBP homologous protein
EGR-1	early growth response transcription factor-1
NF-kB	Nuclear Factor kappa B
GDNF	glial cell-derived neurotrophic factor
GFRAL	GDNF family receptor α-like
RET	rearranged during transfection
ERK	extracellular signal-regulated kinase
PLC1	phosphoinositide phospholipase C 1
PKB	protein kinase B
PI3K	phosphatidylinositol 3-kinase
JNK	c-Jun N-terminal kinase
Bad	Bcl-2-associated death promoter
EGFR	epidermal growth factor receptor
CAS-3	Caspase-3
ELISA	enzyme-linked immunosorbent assay
ECLIA	electrochemiluminescence immunoassay
CLIA	chemiluminescence immunoassay
IVD	in vitro diagnostic
IRMA	immunoradiometric sandwich assay
LC-ESI MS	liquid chromatography electrospray ionization mass spectrometry
NT-proBNP	N-terminal pro-B-type natriuretic peptide
hs-TnT	high-sensitivity troponin T
AHF	acute heart failure
HFH	heart failure hospitalization
CCR2	C-C chemokine receptor 2
VLDL	Very Low-Density Lipoproteins
LDL	Low-Density Lipoproteins
HDL	High-Density Lipoproteins
CAD	coronary artery disease
MI	myocardial infarction
ACS	acute coronary syndrome
hs-CRP	high-sensitivity C-reactive protein
AF	atrial fibrillation
MACE	major adverse cardiac events
AIS	acute ischemic stroke
PSD	post-stroke depression
CABG	coronary artery bypass grafting
SOFA	Sequential Organ Failure Assessment
TAVR	transcatheter aortic valve replacement
sST2	Soluble Suppression of Tumorigenicity 2
